# Social impairment of individuals suffering from different types of chronic orofacial pain

**DOI:** 10.1186/s40510-014-0027-z

**Published:** 2014-04-16

**Authors:** Iacopo Cioffi, Stefania Perrotta, Lucia Ammendola, Roberta Cimino, Stefano Vollaro, Sergio Paduano, Ambrosina Michelotti

**Affiliations:** 1 Department of Neuroscience, University of Naples Federico II, Napoli 80131, Italy; 2 School of Dentistry, University Magna Graecia, Catanzaro 88100, Italy

## Abstract

**Background:**

The daily life of patients suffering from orofacial pain is considerably impaired as compared to healthy subjects. The aim of this study was to investigate the influence of different categories of orofacial pain on the habitual life of adult individuals.

**Methods:**

Seven hundred eighty-one individuals with orofacial pain were recruited from an initial sample of 1,058 patients. All the individuals were allocated to groups according to their diagnosis: myofascial pain (group A, 676 subjects, 525 females and 151 males; mean age ± SD = 35.2 ± 12.6), migraine (group B, 39 subjects, 29 females and 10 males; mean age ± SD 36.0 ± 10.7), and both myofascial pain and migraine (group C, 66 subjects, 56 females and 10 males, mean age ± SD = 35.6 ± 10.8). Characteristic pain intensity (CPI), disability days (DD), disability score (DS), and graded chronic pain intensity (GCPS) were calculated according to Research Diagnostic Criteria for Temporomandibular Disorders (RDC/TMD) axis II. Depression and somatization (nonspecific physical symptoms) scores were also calculated.

**Results:**

A significant association between groups and GCPS categories was found (*p* < 0.0001). *Post hoc* tests showed a significant difference between groups A and B and between A and C, but not between B and C. In group A, the most frequent GCPS score was grade II. The most frequent GCPS score in groups B and C was grade III, indicating a moderate limiting impairment. This score was more frequent in group B (41%) than in the other groups (group A = 20.6%, group C = 34.8%). GCPS grade IV was more frequent in group C (19.7%) than in the other groups. Group C had significantly higher scores for nonspecific physical symptoms than group A (*p* < 0.05).

**Conclusions:**

Myofascial pain and migraine sensibly affect the common daily life of adult individuals. The comorbidity of both conditions determines a major impairment.

## Background

Orofacial pain is relatively frequent in the general population [1]. The role of acute pain is to act as a protector by warning an individual, in order to maintain proper physiological function [2]. It is characterized by a sudden onset, short duration, predictability of treatment outcome, and it is often associated with reversible psychological distress. Differently, chronic pain is defined as pain that persists beyond the usual course of an acute disease process [3], and it is characterized by extended duration. Commonly, it is related to a poorly defined pathology and presents a limited response to medical treatments [3,4].

Chronic pain is often associated with psychological distress and social impairment, with reduced quality of life, working capacity, and social costs [5].

The head and neck region is one of the most common body locations for reported pain [6]. Also, chronic orofacial pain may be associated with social impairment, reduced quality of life, psychological distress, physical disability, reduced economical income, and high costs for the healthcare service [7].

Tempomandibular disorders (TMDs) are regarded as the most common cause of chronic orofacial pain [8]. They comprise a group of disorders that affect the temporomandibular joint (TMJ), the masticatory muscles, or both, and are often associated to musculoskeletal pain, disturbances in mandibular functional movements with impairment of oral function [9-12].

The main reason for patients to seek treatment is that most TMDs are related to the onset of chronic pain [13]. It has been suggested that psychological factors are severely implicated in the initiation as well in the perpetuation of several TMDs [14,15] and that stress, somatic distress, and depression may be potential etiological risk factors for TMD-related pain [16,17]. A comorbidity of TMDs and headaches or migraine has been shown in a number of studies [18-23].

TMDs have been also associated with other chronic pains involving neck, back, and joints. This scenario causes major physical and psychological disability and remarkable health care costs.

Recently, a longitudinal study conducted in the United States, involving thousands of individuals, showed that the comorbidity of TMD pain with other conditions is very frequent and that less than 1% of individuals with TMDs are free of other pain symptoms [22].

The characteristics of chronic TMDs and their related social impairment have been recently investigated in a large international sample, including Italian individuals [24]. However, little is known about which of the different categories of orofacial pain may predominantly affect the habitual life of adult individuals.

The aim of this study was to investigate the pain characteristics and the social impairment determined by muscular TMD-related pain, migraine, and by their comorbidity.

## Methods

Individuals suffering from muscular orofacial pain (myofascial pain or tension type headache), migraine, or both were selected from an initial sample of 1,058 patients referred to the Department of Neuroscience, Section of Orthodontics and Temporomandibular disorders, University of Naples Federico II, Italy. The Research Diagnostic Criteria for TMDs were used to diagnose different subgroups of TMDs [25]. Patients gave their written consent for the collection and analysis of data. The study was approved by the Local Ethical Committee of the Department of Neuroscience, University of Naples Federico II.

The clinical examination was performed by an operator who was calibrated for the assessment of TMDs [26] according to the Research Diagnostic Criteria for Temporomandibular Disorders (RDC/TMD). The clinical examination included the assessment of painful areas that have been subjectively reported by patients, the qualitative assessment (presence of any deviations or deflections) and quantitative analysis of vertical movements of the jaw (open active pain-free, fully open active, passive maximum opening, and evaluation of overbite). Furthermore, joint noises on palpation on vertical movements (clicking or crepitus on opening, closing, or the other way around) and articular noise on palpation during lateralization and protrusion (click or crepitus) were evaluated. Intraoral and extraoral muscle palpation were conducted according to RDC guidelines.

The diagnosis of migraine was performed according to the International Headache Society (IHS) criteria [27]. Each subject had a diagnosis of migraine when reporting at least five headache attacks lasting 4 to 72 h (untreated or unsuccessfully treated) with nausea and/or vomiting or photophobia and phonophobia. Headaches had at least two of the following characteristics: unilateral location, pulsating quality, moderate or severe pain intensity, causing avoidance of routine physical activity (e.g., walking or climbing stairs).

A total of 781 individuals were recruited and allocated to groups according to their diagnosis. Individuals suffering from muscular pain (myofascial pain or tension type headache) were allocated to group A (676 subjects, 525 females and 151 males; mean age ± SD = 35.2 ± 12.6 years), patients with migraine were allocated to group B (39 subjects, 29 females and 10 males; mean age ± SD 36.0 ± 10.7 years), and subjects with both muscular pain and migraine were allocated to group C (66 subjects, 56 females and 10 males; mean age ± SD = 35.6 ± 10.8 years).

All the individuals completed the axis II instrument of RDC/TMD (including the evaluation of depression, nonspecific physical symptoms, and the graded chronic pain scale) [27]. Characteristic pain intensity (CPI), disability days (DD), disability score (DS), and graded chronic pain intensity scores (GCPS) were calculated according to RDC/TMD axis II graded chronic pain scale for all the orofacial pain categories examined. Scores from depression symptoms using checklist 90 (SCL 90) scale for depression and somatization levels (SCL 90 scale for nonspecific physical symptoms) were also computed [28].

### Statistical analysis

The association between groups and GCPS grade categories was evaluated using Kruskal-Wallis test that take into account the ordinal values of the variables. Bonferroni *post hoc* test was used to detect differences between groups.

The analysis of variance was used to assess between-group differences in CPI, DD, and DS. The analyses were performed using SPSS software ver. 20 (IBM Corp., Armonk, NY, USA). The statistical significance was set at *p* < 0.05.

## Results

**Figure 1:**
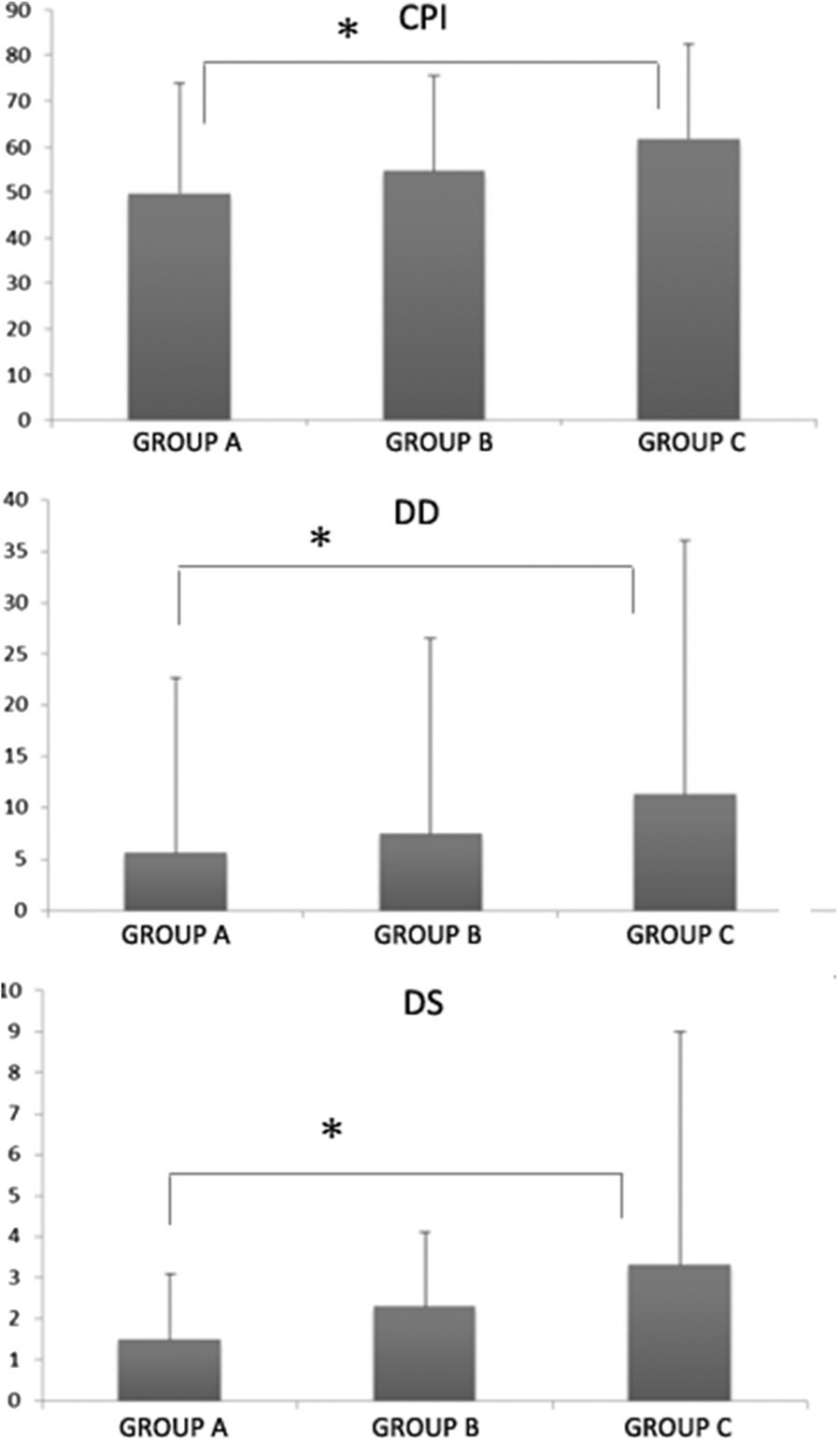
**Mean values for all the groups examined.** CPI, characteristic pain intensity; DD, disability days; DS, disability scores. The bars indicate the standard deviation. Asterisk (*) indicates between-group significant differences (*p* < 0.05).

The CPI, DD, and DS mean values for all the groups examined are reported in Figure 1. Group C had significantly higher scores for CPI (61.8 ± 20.6 vs 49.7 ± 24.0, *p* < 0.001), DD (11.3 ± 24.7 vs 5.64 ± 17.1, *p* = 0.043), and DS (3.3 ± 5.7 vs 1.5 ± 1.6, *p* < 0.001) as compared to group A. A significant association between groups and GCPS categories was found (*p* < 0.0001). The distribution of GCPS scores is reported in Table 1. *Post hoc* tests showed a significant difference between groups A and B and between A and C, but not between B and C.

**Table 1:** Distribution of GCPS scores in the groups examined

	**Grade**				
	**0**	**I**	**II**	**III**	**IV**
Groups					
A	47 (7.0%)	208 (30.8%)	238 (35.2%)	139 (20.6%)	44 (6.5%)
B	1 (2.6%)	9 (23.1%)	8 (20.5%)	16 (41.0%)	5 (12.8%)
C	2 (3.0%)	12 (18.2%)	16 (24.2%)	23 (34.8%)	13 (19.7%)
Between-group significant differences	^a,b^	^a,b^	^a,b^	^a,b^	^a,b^

A significant association between groups and GCPS categories was found (*p* < 0.0001). ^a^Significant difference between groups A and B; ^b^significant difference between A and C.

In group A, the most frequent GCPS score was grade II. The most frequent GCPS score in groups B and C was grade III, indicating a moderate limiting impairment. This score was more frequent in group B (41%) than in the other groups (group A = 20.6%, group C = 34.8%). GCPS grade IV was more frequent in group C (19.7%) than in the other groups. No significant differences between groups were found with respect to depression scores. Group C had significantly higher scores for nonspecific physical symptoms than group A (*p* < 0.05).

## Discussion

Chronic pain is associated with severe and extensive psychological, social, and economic factors, with high demand on the health services, particularly primary care [29].

Studies measuring the impact of chronic pain on individual life and health tended to focus on specific conditions or groups of conditions with pain comorbidity [29].

Chronic orofacial pain has a major impact on the quality of life of people who suffer from TMDs [22,30,31], headache, and migraine [23,32].

There is evidence that TMD pain has a detrimental effect on physical health daily activity, psychological health, employment, and economic well-being [30].

The results of the current study showed that individuals suffering from migraine had more frequent social impairment as compared to TMD patients, who also present disability in accordance to a previous study [24]. This result is likely related to the characteristics of pain that has been reported to be severe in headache and migraine individuals [33,34] as compared to TMD patients that commonly suffer from moderate to severe pain [24,34].

In this study, the comorbidity of migraine and TMD muscular pain determined a significant social impairment, and it is significantly more unfavorable than the singular disturbances.

A possible relation between temporomandibular disorders and headaches has been investigated by some authors [35-38]. Possible pathogenetic mechanisms have also been proposed. It has been suggested that active trigger points are highly prevalent in individuals suffering from tension-type headache or migraine and that their stimulation may induce typical headache symptoms. Furthermore, patients with headaches and migraine present an increased tenderness of pericranial tissues and muscles [39].

The possible relation between migraine and myofascial pain has been matter of debate [40-42]. It is possible that the activation of peripheral nociceptive stimuli through trigger points may lead to the activation of trigeminovascular system, via nucleus caudalis, in predisposed individuals, determining a migraine attack [42].

It has been shown that depressive and anxiety symptoms are risk factors for temporomandibular pain [17]. In this study, individuals suffering from orofacial pain had moderate depression and somatization symptoms. This was also reported by Manfredini and coworkers [33] who found moderate to severe somatization and depression in individuals suffering from TMDs.

## Conclusions

Our findings reveal that individuals with comorbidity of migraine and muscular TMD pain present a very important social impairment. Also, the comorbid pain is much more unfavorable than the singular disturbances. The comorbidity of migraine and muscular pain influences the psychological status of the individuals, determining higher somatization scores. This, in turn, could sensibly affect the therapeutical approach of these patients and affect the treatment outcomes. For these reasons, clinicians should be aware of collecting adequate anamnestic data since TMD patients often do not report comorbid pain on first referral. These patients should be screened for psychological distress and evaluated by physicians for their main pain complaints.

## Competing interests

The authors declare that they have no competing interests.

## Authors’ contributions

IC and AM designed the study and are responsible for the research protocol adopted. They coordinated the study. The statistical analysis was performed by IC. SP, LA, SP and RC managed the clinical phase of this research study and were responsible for the allocation to groups. SV was responsible for the clinical examination of the patients. All authors read and approved the final manuscript.
